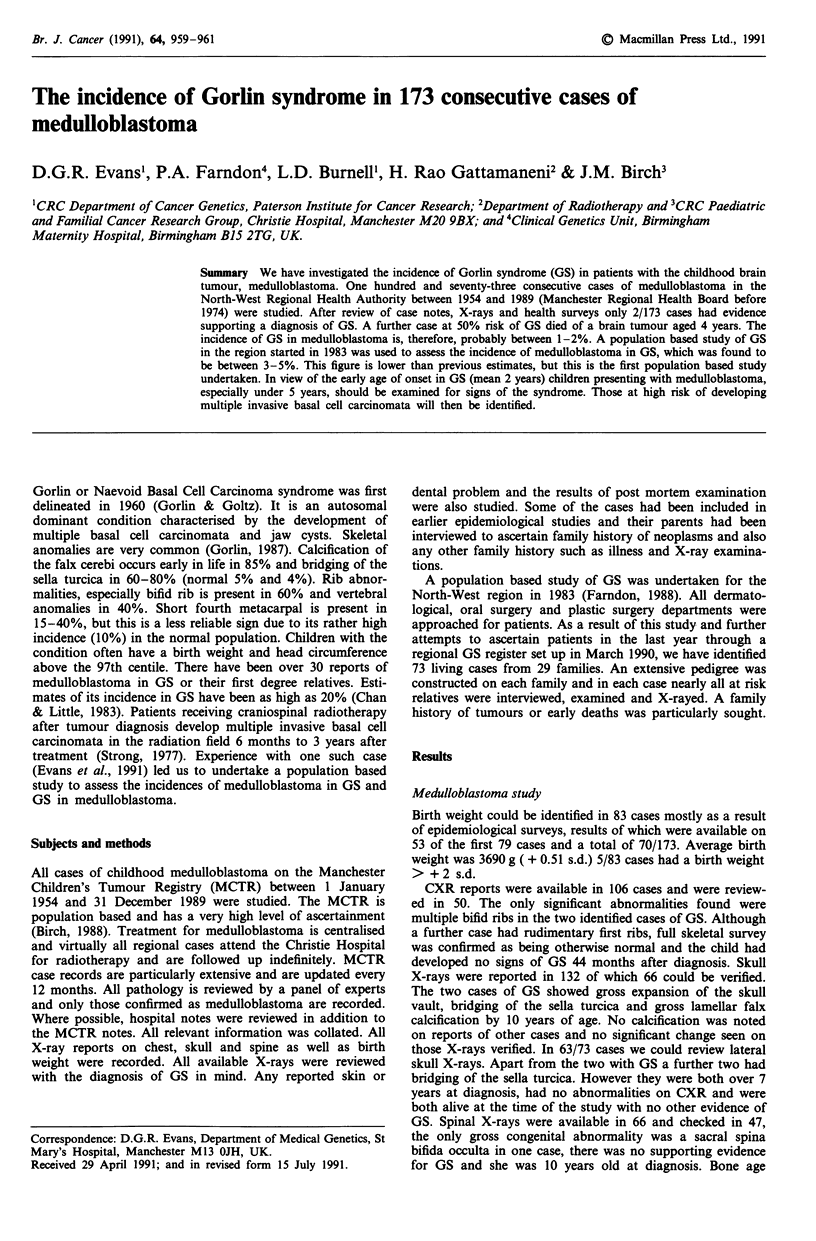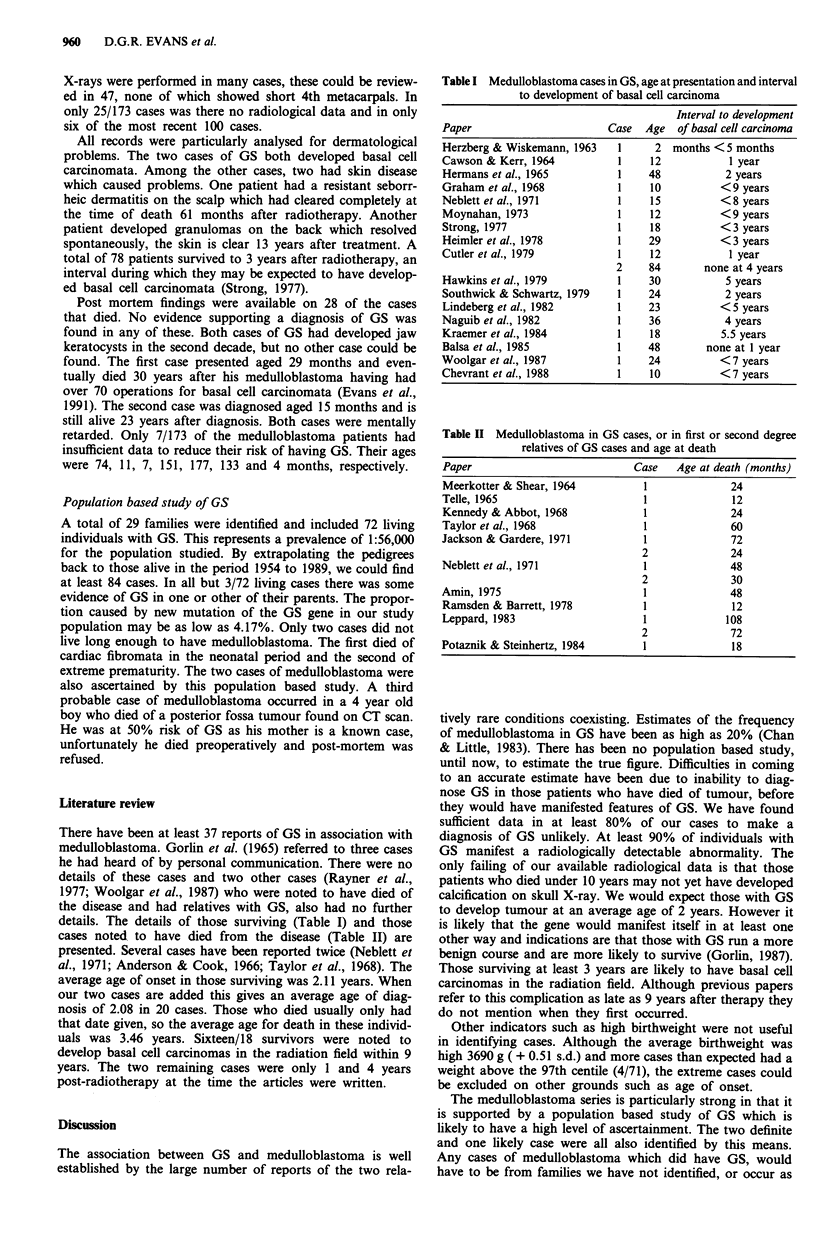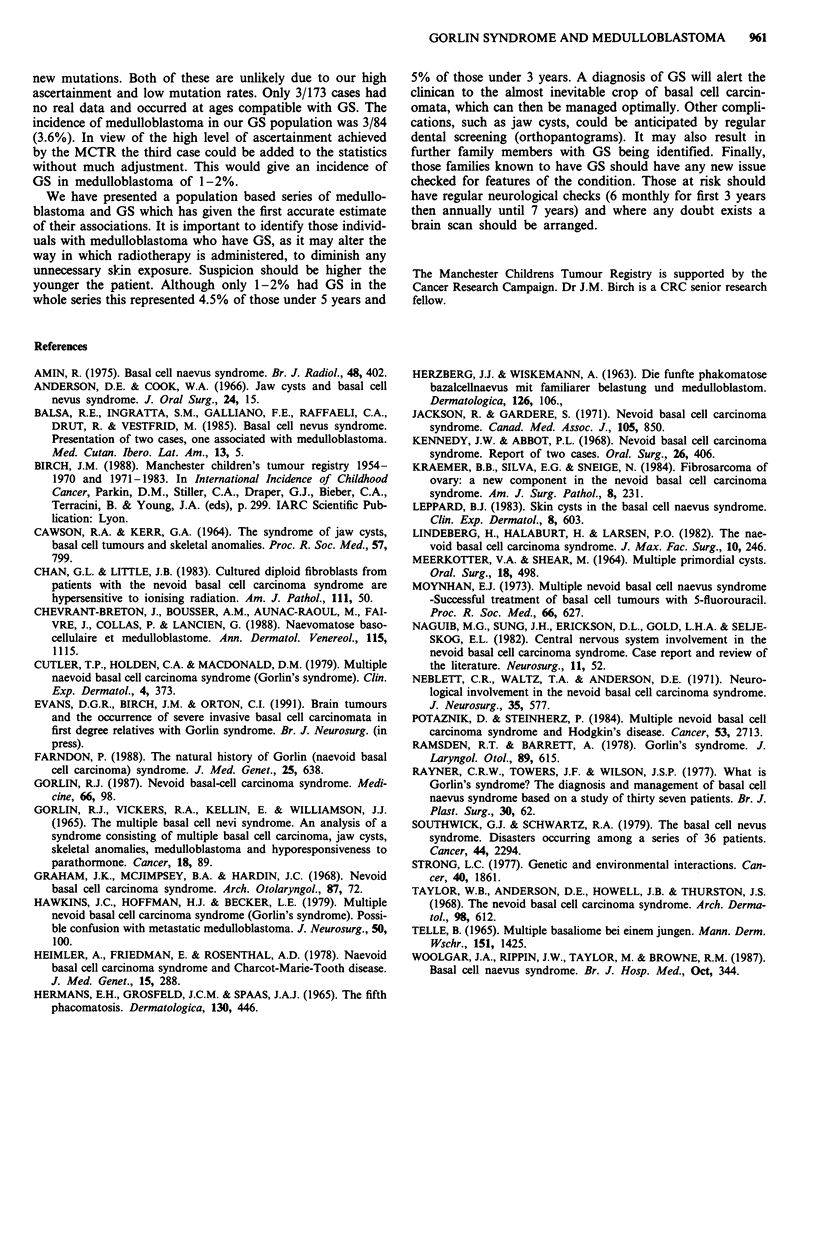# The incidence of Gorlin syndrome in 173 consecutive cases of medulloblastoma.

**DOI:** 10.1038/bjc.1991.435

**Published:** 1991-11

**Authors:** D. G. Evans, P. A. Farndon, L. D. Burnell, H. R. Gattamaneni, J. M. Birch

**Affiliations:** CRC Department of Cancer Genetics, Paterson Institute for Cancer Research, Manchester, UK.

## Abstract

We have investigated the incidence of Gorlin syndrome (GS) in patients with the childhood brain tumour, medulloblastoma. One hundred and seventy-three consecutive cases of medulloblastoma in the North-West Regional Health Authority between 1954 and 1989 (Manchester Regional Health Board before 1974) were studied. After review of case notes, X-rays and health surveys only 2/173 cases had evidence supporting a diagnosis of GS. A further case at 50% risk of GS died of a brain tumour aged 4 years. The incidence of GS in medulloblastoma is, therefore, probably between 1-2%. A population based study of GS in the region started in 1983 was used to assess the incidence of medulloblastoma in GS, which was found to be between 3-5%. This figure is lower than previous estimates, but this is the first population based study undertaken. In view of the early age of onset in GS (mean 2 years) children presenting with medulloblastoma, especially under 5 years, should be examined for signs of the syndrome. Those at high risk of developing multiple invasive basal cell carcinomata will then be identified.


Br. J. Cancer (1991), 64, 959-961 © Macmillan Press Ltd., 1991

### The incidence of Gorlin syndrome in 173 consecutive cases of medulloblastoma

D.G.R. Evans[1], P.A. Farndon[4], L.D. Burnell[1], H. Rao Gattamaneni[2] & J.M. Birch[3]

[1]*CRC Department of Cancer Genetics, Paterson Institute for Cancer Research;* [2]*Department of Radiotherapy and* [3]*CRC Paediatric and Familial Cancer Research Group, Christie Hospital, Manchester M20 9BX; and* [4]*Clinical Genetics Unit, Birmingham Maternity Hospital, Birmingham B15 2TG, UK.*

**Summary** We have investigated the incidence of Gorlin syndrome (GS) in patients with the childhood brain tumour, medulloblastoma. One hundred and seventy-three consecutive cases of medulloblastoma in the North-West Regional Health Authority between 1954 and 1989 (Manchester Regional Health Board before 1974) were studied. After review of case notes, X-rays and health surveys only 2/173 cases had evidence supporting a diagnosis of GS. A further case at 50% risk of GS died of a brain tumour aged 4 years. The incidence of GS in medulloblastoma is, therefore, probably between 1–2%. A population based study of GS in the region started in 1983 was used to assess the incidence of medulloblastoma in GS, which was found to be between 3–5%. This figure is lower than previous estimates, but this is the first population based study undertaken. In view of the early age of onset in GS (mean 2 years) children presenting with medulloblastoma, especially under 5 years, should be examined for signs of the syndrome. Those at high risk of developing multiple invasive basal cell carcinomata will then be identified.

Gorlin or Naevoid Basal Cell Carcinoma syndrome was first delineated in 1960 (Gorlin & Goltz). It is an autosomal dominant condition characterised by the development of multiple basal cell carcinomata and jaw cysts. Skeletal anomalies are very common (Gorlin, 1987). Calcification of the falx cerebi occurs early in life in 85% and bridging of the sella turcica in 60–80% (normal 5% and 4%). Rib abnormalities, especially bifid rib is present in 60% and vertebral anomalies in 40%. Short fourth metacarpal is present in 15–40%, but this is a less reliable sign due to its rather high incidence (10%) in the normal population. Children with the condition often have a birth weight and head circumference above the 97th centile. There have been over 30 reports of medulloblastoma in GS or their first degree relatives. Estimates of its incidence in GS have been as high as 20% (Chan & Little, 1983). Patients receiving craniospinal radiotherapy after tumour diagnosis develop multiple invasive basal cell carcinomata in the radiation field 6 months to 3 years after treatment (Strong, 1977). Experience with one such case (Evans *et al.*, 1991) led us to undertake a population based study to assess the incidences of medulloblastoma in GS and GS in medulloblastoma.

### Subjects and methods

All cases of childhood medulloblastoma on the Manchester Children's Tumour Registry (MCTR) between 1 January 1954 and 31 December 1989 were studied. The MCTR is population based and has a very high level of ascertainment (Birch, 1988). Treatment for medulloblastoma is centralised and virtually all regional cases attend the Christie Hospital for radiotherapy and are followed up indefinitely. MCTR case records are particularly extensive and are updated every 12 months. All pathology is reviewed by a panel of experts and only those confirmed as medulloblastoma are recorded. Where possible, hospital notes were reviewed in addition to the MCTR notes. All relevant information was collated. All X-ray reports on chest, skull and spine as well as birth weight were recorded. All available X-rays were reviewed with the diagnosis of GS in mind. Any reported skin or dental problem and the results of post mortem examination were also studied. Some of the cases had been included in earlier epidemiological studies and their parents had been interviewed to ascertain family history of neoplasms and also any other family history such as illness and X-ray examinations.

A population based study of GS was undertaken for the North-West region in 1983 (Farndon, 1988). All dermatological, oral surgery and plastic surgery departments were approached for patients. As a result of this study and further attempts to ascertain patients in the last year through a regional GS register set up in March 1990, we have identified 73 living cases from 29 families. An extensive pedigree was constructed on each family and in each case nearly all at risk relatives were interviewed, examined and X-rayed. A family history of tumours or early deaths was particularly sought.

### Results

### *Medulloblastoma study*

Birth weight could be identified in 83 cases mostly as a result of epidemiological surveys, results of which were available on 53 of the first 79 cases and a total of 70/173. Average birth weight was 3690 g ( + 0.51 s.d.) 5/83 cases had a birth weight > + 2 s.d.

CXR reports were available in 106 cases and were reviewed in 50. The only significant abnormalities found were multiple bifid ribs in the two identified cases of GS. Although a further case had rudimentary first ribs, full skeletal survey was confirmed as being otherwise normal and the child had developed no signs of GS 44 months after diagnosis. Skull X-rays were reported in 132 of which 66 could be verified. The two cases of GS showed gross expansion of the skull vault, bridging of the sella turcica and gross lamellar falx calcification by 10 years of age. No calcification was noted on reports of other cases and no significant change seen on those X-rays verified. In 63/73 cases we could review lateral skull X-rays. Apart from the two with GS a further two had bridging of the sella turcica. However they were both over 7 years at diagnosis, had no abnormalities on CXR and were both alive at the time of the study with no other evidence of GS. Spinal X-rays were available in 66 and checked in 47, the only gross congenital abnormality was a sacral spina bifida occulta in one case, there was no supporting evidence for GS and she was 10 years old at diagnosis. Bone age

Correspondence: D.G.R. Evans, Department of Medical Genetics, St Mary's Hospital, Manchester M13 0JH, UK.
Received 29 April 1991; and in revised form 15 July 1991.

X-rays were performed in many cases, these could be reviewed in 47, none of which showed short 4th metacarpals. In only 25/173 cases was there no radiological data and in only six of the most recent 100 cases.

All records were particularly analysed for dermatological problems. The two cases of GS both developed basal cell carcinomata. Among the other cases, two had skin disease which caused problems. One patient had a resistant seborrheic dermatitis on the scalp which had cleared completely at the time of death 61 months after radiotherapy. Another patient developed granulomas on the back which resolved spontaneously, the skin is clear 13 years after treatment. A total of 78 patients survived to 3 years after radiotherapy, an interval during which they may be expected to have developed basal cell carcinomata (Strong, 1977).

Post mortem findings were available on 28 of the cases that died. No evidence supporting a diagnosis of GS was found in any of these. Both cases of GS had developed jaw keratocysts in the second decade, but no other case could be found. The first case presented aged 29 months and eventually died 30 years after his medulloblastoma having had over 70 operations for basal cell carcinomata (Evans *et al.*, 1991). The second case was diagnosed aged 15 months and is still alive 23 years after diagnosis. Both cases were mentally retarded. Only 7/173 of the medulloblastoma patients had insufficient data to reduce their risk of having GS. Their ages were 74, 11, 7, 151, 177, 133 and 4 months, respectively.

*Population based study of GS*

A total of 29 families were identified and included 72 living individuals with GS. This represents a prevalence of 1:56,000 for the population studied. By extrapolating the pedigrees back to those alive in the period 1954 to 1989, we could find at least 84 cases. In all but 3/72 living cases there was some evidence of GS in one or other of their parents. The proportion caused by new mutation of the GS gene in our study population may be as low as 4.17%. Only two cases did not live long enough to have medulloblastoma. The first died of cardiac fibromata in the neonatal period and the second of extreme prematurity. The two cases of medulloblastoma were also ascertained by this population based study. A third probable case of medulloblastoma occurred in a 4 year old boy who died of a posterior fossa tumour found on CT scan. He was at 50% risk of GS as his mother is a known case, unfortunately he died preoperatively and post-mortem was refused.

### Literature review

There have been at least 37 reports of GS in association with medulloblastoma. Gorlin *et al.* (1965) referred to three cases he had heard of by personal communication. There were no details of these cases and two other cases (Rayner *et al.*, 1977; Woolgar *et al.*, 1987) who were noted to have died of the disease and had relatives with GS, also had no further details. The details of those surviving (Table I) and those cases noted to have died from the disease (Table II) are presented. Several cases have been reported twice (Neblett *et al.*, 1971; Anderson & Cook, 1966; Taylor *et al.*, 1968). The average age of onset in those surviving was 2.11 years. When our two cases are added this gives an average age of diagnosis of 2.08 in 20 cases. Those who died usually only had that date given, so the average age for death in these individuals was 3.46 years. Sixteen/18 survivors were noted to develop basal cell carcinomas in the radiation field within 9 years. The two remaining cases were only 1 and 4 years post-radiotherapy at the time the articles were written.

### Discussion

The association between GS and medulloblastoma is well established by the large number of reports of the two relatively rare conditions coexisting. Estimates of the frequency of medulloblastoma in GS have been as high as 20% (Chan & Little, 1983). There has been no population based study, until now, to estimate the true figure. Difficulties in coming to an accurate estimate have been due to inability to diagnose GS in those patients who have died of tumour, before they would have manifested features of GS. We have found sufficient data in at least 80% of our cases to make a diagnosis of GS unlikely. At least 90% of individuals with GS manifest a radiologically detectable abnormality. The only failing of our available radiological data is that those patients who died under 10 years may not yet have developed calcification on skull X-ray. We would expect those with GS to develop tumour at an average age of 2 years. However it is likely that the gene would manifest itself in at least one other way and indications are that those with GS run a more benign course and are more likely to survive (Gorlin, 1987). Those surviving at least 3 years are likely to have basal cell carcinomas in the radiation field. Although previous papers refer to this complication as late as 9 years after therapy they do not mention when they first occurred.

Other indicators such as high birthweight were not useful in identifying cases. Although the average birthweight was high 3690 g ( + 0.51 s.d.) and more cases than expected had a weight above the 97th centile (4/71), the extreme cases could be excluded on other grounds such as age of onset.

The medulloblastoma series is particularly strong in that it is supported by a population based study of GS which is likely to have a high level of ascertainment. The two definite and one likely case were all also identified by this means. Any cases of medulloblastoma which did have GS, would have to be from families we have not identified, or occur as

**Table I** Medulloblastoma cases in GS, age at presentation and interval to development of basal cell carcinoma

| *Paper* | *Case* | *Age* | *Interval to development of basal cell carcinoma* |
|---|---|---|---|
| Herzberg & Wiskemann, 1963 | 1 | 2 | months < 5 months |
| Cawson & Kerr, 1964 | 1 | 12 | 1 year |
| Hermans *et al.*, 1965 | 1 | 48 | 2 years |
| Graham *et al.*, 1968 | 1 | 10 | < 9 years |
| Neblett *et al.*, 1971 | 1 | 15 | < 8 years |
| Moynahan, 1973 | 1 | 12 | < 9 years |
| Strong, 1977 | 1 | 18 | < 3 years |
| Heimler *et al.*, 1978 | 1 | 29 | < 3 years |
| Cutler *et al.*, 1979 | 1 | 12 | 1 year |
| | 2 | 84 | none at 4 years |
| Hawkins *et al.*, 1979 | 1 | 30 | 5 years |
| Southwick & Schwartz, 1979 | 1 | 24 | 2 years |
| Lindeberg *et al.*, 1982 | 1 | 23 | < 5 years |
| Naguib *et al.*, 1982 | 1 | 36 | 4 years |
| Kraemer *et al.*, 1984 | 1 | 18 | 5.5 years |
| Balsa *et al.*, 1985 | 1 | 48 | none at 1 year |
| Woolgar *et al.*, 1987 | 1 | 24 | < 7 years |
| Chevrant *et al.*, 1988 | 1 | 10 | < 7 years |

**Table II** Medulloblastoma in GS cases, or in first or second degree relatives of GS cases and age at death

| *Paper* | *Case* | *Age at death (months)* |
|---|---|---|
| Meerkotter & Shear, 1964 | 1 | 24 |
| Telle, 1965 | 1 | 12 |
| Kennedy & Abbot, 1968 | 1 | 24 |
| Taylor *et al.*, 1968 | 1 | 60 |
| Jackson & Gardere, 1971 | 1 | 72 |
| | 2 | 24 |
| Neblett *et al.*, 1971 | 1 | 48 |
| | 2 | 30 |
| Amin, 1975 | 1 | 48 |
| Ramsden & Barrett, 1978 | 1 | 12 |
| Leppard, 1983 | 1 | 108 |
| | 2 | 72 |
| Potaznik & Steinhertz, 1984 | 1 | 18 |

new mutations. Both of these are unlikely due to our high ascertainment and low mutation rates. Only 3/173 cases had no real data and occurred at ages compatible with GS. The incidence of medulloblastoma in our GS population was 3/84 (3.6%). In view of the high level of ascertainment achieved by the MCTR the third case could be added to the statistics without much adjustment. This would give an incidence of GS in medulloblastoma of 1–2%.

We have presented a population based series of medulloblastoma and GS which has given the first accurate estimate of their associations. It is important to identify those individuals with medulloblastoma who have GS, as it may alter the way in which radiotherapy is administered, to diminish any unnecessary skin exposure. Suspicion should be higher the younger the patient. Although only 1–2% had GS in the whole series this represented 4.5% of those under 5 years and 5% of those under 3 years. A diagnosis of GS will alert the clinican to the almost inevitable crop of basal cell carcinomata, which can then be managed optimally. Other complications, such as jaw cysts, could be anticipated by regular dental screening (orthopantograms). It may also result in further family members with GS being identified. Finally, those families known to have GS should have any new issue checked for features of the condition. Those at risk should have regular neurological checks (6 monthly for first 3 years then annually until 7 years) and where any doubt exists a brain scan should be arranged.

The Manchester Childrens Tumour Registry is supported by the Cancer Research Campaign. Dr J.M. Birch is a CRC senior research fellow.

### References

AMIN, R. (1975). Basal cell naevus syndrome. *Br. J. Radiol.*, **48**, 402.

ANDERSON, D.E. & COOK, W.A. (1966). Jaw cysts and basal cell nevus syndrome. *J. Oral Surg.*, **24**, 15.

BALSA, R.E., INGRATTA, S.M., GALLIANO, F.E., RAFFAELI, C.A., DRUT, R. & VESTFRID, M. (1985). Basal cell nevus syndrome. Presentation of two cases, one associated with medulloblastoma. *Med. Cutan. Ibero. Lat. Am.*, **13**, 5.

BIRCH, J.M. (1988). Manchester children's tumour registry 1954–1970 and 1971–1983. In *International Incidence of Childhood Cancer*, Parkin, D.M., Stiller, C.A., Draper, G.J., Bieber, C.A., Terracini, B. & Young, J.A. (eds), p. 299. IARC Scientific Publication: Lyon.

CAWSON, R.A. & KERR, G.A. (1964). The syndrome of jaw cysts, basal cell tumours and skeletal anomalies. *Proc. R. Soc. Med.*, **57**, 799.

CHAN, G.L. & LITTLE, J.B. (1983). Cultured diploid fibroblasts from patients with the nevoid basal cell carcinoma syndrome are hypersensitive to ionising radiation. *Am. J. Pathol.*, **111**, 50.

CHEVRANT-BRETON, J., BOUSSER, A.M., AUNAC-RAOUL, M., FAIVRE, J., COLLAS, P. & LANCIEN, G. (1988). Naevomatose basocellulaire et medulloblastome. *Ann. Dermatol. Venereol.*, **115**, 1115.

CUTLER, T.P., HOLDEN, C.A. & MACDONALD, D.M. (1979). Multiple naevoid basal cell carcinoma syndrome (Gorlin's syndrome). *Clin. Exp. Dermatol.*, **4**, 373.

EVANS, D.G.R., BIRCH, J.M. & ORTON, C.I. (1991). Brain tumours and the occurrence of severe invasive basal cell carcinomata in first degree relatives with Gorlin syndrome. *Br. J. Neurosurg.* (in press).

FARNDON, P. (1988). The natural history of Gorlin (naevoid basal cell carcinoma) syndrome. *J. Med. Genet.*, **25**, 638.

GORLIN, R.J. (1987). Nevoid basal-cell carcinoma syndrome. *Medicine*, **66**, 98.

GORLIN, R.J., VICKERS, R.A., KELLIN, E. & WILLIAMSON, J.J. (1965). The multiple basal cell nevi syndrome. An analysis of a syndrome consisting of multiple basal cell carcinoma, jaw cysts, skeletal anomalies, medulloblastoma and hyporesponsiveness to parathormone. *Cancer*, **18**, 89.

GRAHAM, J.K., MCJIMPSEY, B.A. & HARDIN, J.C. (1968). Nevoid basal cell carcinoma syndrome. *Arch. Otolaryngol.*, **87**, 72.

HAWKINS, J.C., HOFFMAN, H.J. & BECKER, L.E. (1979). Multiple nevoid basal cell carcinoma syndrome (Gorlin's syndrome). Possible confusion with metastatic medulloblastoma. *J. Neurosurg.*, **50**, 100.

HEIMLER, A., FRIEDMAN, E. & ROSENTHAL, A.D. (1978). Naevoid basal cell carcinoma syndrome and Charcot-Marie-Tooth disease. *J. Med. Genet.*, **15**, 288.

HERMANS, E.H., GROSFELD, J.C.M. & SPAAS, J.A.J. (1965). The fifth phacomatosis. *Dermatologica*, **130**, 446.

HERZBERG, J.J. & WISKEMANN, A. (1963). Die funfte phakomatose bazalcellnaevus mit familiarer belastung und medulloblastom. *Dermatologica*, **126**, 106.,

JACKSON, R. & GARDERE, S. (1971). Nevoid basal cell carcinoma syndrome. *Canad. Med. Assoc. J.*, **105**, 850.

KENNEDY, J.W. & ABBOT, P.L. (1968). Nevoid basal cell carcinoma syndrome. Report of two cases. *Oral. Surg.*, **26**, 406.

KRAEMER, B.B., SILVA, E.G. & SNEIGE, N. (1984). Fibrosarcoma of ovary: a new component in the nevoid basal cell carcinoma syndrome. *Am. J. Surg. Pathol.*, **8**, 231.

LEPPARD, B.J. (1983). Skin cysts in the basal cell naevus syndrome. *Clin. Exp. Dermatol.*, **8**, 603.

LINDEBERG, H., HALABURT, H. & LARSEN, P.O. (1982). The naevoid basal cell carcinoma syndrome. *J. Max. Fac. Surg.*, **10**, 246.

MEERKOTTER, V.A. & SHEAR, M. (1964). Multiple primordial cysts. *Oral. Surg.*, **18**, 498.

MOYNHAN, E.J. (1973). Multiple nevoid basal cell naevus syndrome -Successful treatment of basal cell tumours with 5-fluorouracil. *Proc. R. Soc. Med.*, **66**, 627.

NAGUIB, M.G., SUNG, J.H., ERICKSON, D.L., GOLD, L.H.A. & SELJESKOG, E.L. (1982). Central nervous system involvement in the nevoid basal cell carcinoma syndrome. Case report and review of the literature. *Neurosurg.*, **11**, 52.

NEBLETT, C.R., WALTZ, T.A. & ANDERSON, D.E. (1971). Neurological involvement in the nevoid basal cell carcinoma syndrome. *J. Neurosurg.*, **35**, 577.

POTAZNIK, D. & STEINHERZ, P. (1984). Multiple nevoid basal cell carcinoma syndrome and Hodgkin's disease. *Cancer*, **53**, 2713.

RAMSDEN, R.T. & BARRETT, A. (1978). Gorlin's syndrome. *J. Laryngol. Otol.*, **89**, 615.

RAYNER, C.R.W., TOWERS, J.F. & WILSON, J.S.P. (1977). What is Gorlin's syndrome? The diagnosis and management of basal cell naevus syndrome based on a study of thirty seven patients. *Br. J. Plast. Surg.*, **30**, 62.

SOUTHWICK, G.J. & SCHWARTZ, R.A. (1979). The basal cell nevus syndrome. Disasters occurring among a series of 36 patients. *Cancer*, **44**, 2294.

STRONG, L.C. (1977). Genetic and environmental interactions. *Cancer*, **40**, 1861.

TAYLOR, W.B., ANDERSON, D.E., HOWELL, J.B. & THURSTON, J.S. (1968). The nevoid basal cell carcinoma syndrome. *Arch. Dermatol.*, **98**, 612.

TELLE, B. (1965). Multiple basaliome bei einem jungen. *Mann. Derm. Wschr.*, **151**, 1425.

WOOLGAR, J.A., RIPPIN, J.W., TAYLOR, M. & BROWNE, R.M. (1987). Basal cell naevus syndrome. *Br. J. Hosp. Med.*, **Oct**, 344.